# Beyond efficacy parity: a novel cost-equilibrium framework for value assessment of competing third-line therapies in metastatic colorectal cancer

**DOI:** 10.3389/fphar.2025.1606742

**Published:** 2025-08-14

**Authors:** Shunlong Ou, Bingjie Wang, Jing Luo, Qian Jiang

**Affiliations:** ^1^ Department of Pharmacy, Sichuan Clinical Research Center for Cancer, Sichuan Cancer Hospital and Institute, Sichuan Cancer Center, University of Electronic Science and Technology of China, Chengdu, China; ^2^ Faculty of Science and Technology University of Macau, Macau, China; ^3^ Department of Pharmacy, The Second People’s Hospital of Yibin, Yibin, China

**Keywords:** fruquintinib, regorafenib, cost, equilibrium, mathematical model

## Abstract

**Background:**

Colorectal cancer remains a leading cause of global cancer mortality, with metastatic CRC (mCRC) requiring sequential therapies after first line treatment failure. While regorafenib and fruquintinib are guideline-endorsed third-line options, their comparative value remains unestablished due to absent head-to-head trials. This real-world study evaluates clinical outcomes, safety, and cost differentials to model value-equilibrium pricing.

**Methods:**

A retrospective cohort analysis included 25 mCRC patients (regorafenib: n = 5; fruquintinib: n = 20) treated at Sichuan Cancer Hospital (2021–2022) with follow-up through June 2023. Outcomes included real-world disease control rate (rwDCR), adverse events (CTCAE v4.03-graded), and daily treatment costs (medication, dose adjustments, adverse event management). A Monte Carlo simulation modeled cost equilibrium using Generalized Beta Distribution-derived adverse event variability.

**Results:**

Baseline characteristics were balanced (median age: 58–63; 60%–70% male). rwDCR showed no significant difference (20% vs 25%, p = 1.000). Regorafenib demonstrated higher grade 3–4 toxicities (60.0% vs 20.0%), including hepatotoxicity (40.0% vs 15.0%) and hand-foot skin reaction (20.0% vs 0%). Fruquintinib exhibited unique hypertension (10.0%) and proteinuria (20.0%). Regorafenib incurred 75% higher daily costs (¥455.53 vs ¥259.96, p = 0.001), primarily from medication expenses (¥439.82 vs ¥253.71, p = 0.014). Pharmacoeconomic modeling identified regorafenib’s value-based pricing threshold at 47.35% of current costs (¥248.03/day; 95% CI: 247.98–248.09), revealing a 111% price-to-value mismatch.

**Conclusion:**

Fruquintinib demonstrates comparable efficacy with superior safety and cost-effectiveness in third-line mCRC. Regorafenib’s pricing exceeds its clinical value by twofold, underscoring systemic misalignment between drug costs and therapeutic benefit. These findings advocate for value-driven pricing reforms integrating toxicity-related economic burdens and provide a replicable framework for indirect treatment comparisons in oncology. However, the small sample size reduced statistical power, potentially biasing the findings.

## Introduction

Colorectal cancer (CRC) remains a critical global health burden, ranking as the third most diagnosed malignancy (1.9 million new cases) and the second leading cause of cancer-related mortality (0.9 million deaths) worldwide in 2022 (Bray et al., 2024). Metastatic progression occurs in 25% of CRC patients at initial diagnosis and affects over 50% during therapeutic interventions (Van Cutsem et al., 2013; Zhang et al., 2020). Current first-line treatment for metastatic CRC (mCRC) involves fluoropyrimidine-based regimens with leucovorin, supplemented by oxaliplatin or irinotecan, (de Gramont et al., 2023; Douillard et al., 2000), often combined with targeted agents like bevacizumab or cetuximab to enhance clinical outcomes (Saltz et al., 2023; Heinemann et al., 2014). Despite these advancements, a substantial proportion of mCRC patients experience disease progression following second-line chemotherapy, necessitating third-line therapeutic strategies. Landmark trials including CORRECT, CONCUR, and FRESCO established regorafenib and fruquintinib as guideline-recommended third-line options, (Grothey et al., 2013; Li et al., 2015; Li et al., 2018), with subsequent incorporation into clinical practice guidelines (Benson et al., 2017; Shir and ley, 2018).

Notably, pivotal trials for both agents employed placebo-controlled designs rather than direct comparator studies, (Grothey et al., 2013; Li et al., 2015; Li et al., 2018), resulting in an evidence gap regarding their comparative therapeutic value (Zhang et al., 2020; Jing et al., 2019). While network meta-analysis offers methodological advantages for indirect treatment comparisons through quantitative outcome ranking^,^ (Song et al., 2009), this approach remains constrained by inherent assumptions and primarily addresses efficacy/safety parameters rather than multidimensional value assessments. Recent initiatives by leading oncology societies—including the European Society for Medical Oncology (ESMO) and American Society of Clinical Oncology (ASCO)—have developed value frameworks to evaluate anticancer therapies across multiple dimensions (Karas et al., 2021). However, these frameworks predominantly rely on randomized controlled trial data, limiting their utility for direct cost-value comparisons between regorafenib and fruquintinib in the absence of head-to-head clinical evidence.

A critical paradox persists in public perceptions of anticancer drug pricing: while societal narratives often attribute high costs to research and development expenditures, empirical evidence indicates that government-funded academic institutions constitute the primary source of pharmaceutical innovation (Kesselheim et al., 2015). Patients increasingly expect therapeutic costs to align with clinical value creation, yet multiple studies demonstrate a concerning dissociation between anticancer drug pricing and measurable clinical benefits (Vivot et al., 2017; Jiang et al., 2020; Vokinger et al., 2020). This discrepancy underscores the urgent need for value-based pricing models, particularly for therapeutic alternatives with overlapping indications but divergent cost structures.

Building upon prior comparative analyses demonstrating comparable efficacy but distinct safety profiles between regorafenib and fruquintinib (Zhang et al., 2020; Jing et al., 2019). this investigation positions regorafenib (higher-cost alternative) against fruquintinib (lower-cost reference) under the pharmacoeconomic premise of therapeutic equivalence. Recognizing differential adverse event management costs as the primary value determinant, we developed a novel pharmacoeconomic model to quantify cost differential equilibrium between these agents. This methodology enables derivation of regorafenib’s value-based alternative pricing threshold relative to fruquintinib’s established cost structure.

## Methods

### Study design and patients

This retrospective single-center investigation de-identified patient data extracted from electronic medical records. The study design and reporting framework rigorously adhered to STROBE (Strengthening the Reporting of Observational Studies in Epidemiology) guidelines for observational research (von Elm et al., 2014). The study protocol was reviewed and approved by the Institutional Review Board of Sichuan Cancer Hospital in Chengdu, China (approval number: SCCEC-02-2023-006) on 13 January 2023. Based on strict patient privacy protection measures, including the use of de-identified data with unique study codes replacing direct patient identifiers (e.g., names and medical record numbers used only for linkage purposes internally, not in the analysis dataset), and the observational study design, the Institutional Review Board granted formal authorization for waiver of informed consent.

This study included colorectal cancer patients treated with regorafenib or fruquintinib monotherapy between 1 January 2021 and 31 December 2022. The inclusion criteria were: (1) Pathologically confirmed metastatic colorectal cancer; (2) Previous treatment with fluorouracil-, oxaliplatin-, and irinotecan-based chemotherapy regimens, with prior exposure or ineligibility for anti-VEGF or anti-EGFR therapies; (3) Aged 18–75 years. Exclusion criteria were: (1) Incomplete medical records; (2) Concurrent administration of other chemotherapeutic agents; (3) Lost to follow-up.

### Endpoint definition

The primary endpoint of this study was safety evaluation, with secondary endpoint of real-world disease control rate (rwDCR). Adverse events (AEs) were assessed and graded according to the National Cancer Institute’s Common Terminology Criteria for Adverse Events version 4.03. rwDCR was determined as the proportion of patients achieving complete response (CR), partial response (PR), or maintaining stable disease (SD) and operationalized using modified Response Evaluation Criteria in Solid Tumor (RECIST) 1.1 criteria adapted for real-world data. Serial telephone follow-up assessments were conducted at 3-month intervals during the observation period (March and June 2023), with a final data cutoff date of 30 June 2023. All outcome measures were analyzed using intention-to-treat principles.

### Data extraction

Demographic and clinical characteristic data were collected during the baseline period, including primary tumor site, metastatic lesion locations, types of systemic therapies and the lines of therapy. Clinical endpoints available in the electronic medical records were assessed in the follow-up, encompassing real-world best tumor response during treatment. This metric integrates clinician-evaluated clinical notes with radiologic assessments from imaging reports (Hebart et al., 2019; Ivanova et al., 2017). Two researchers (SL O, QJ) independently performed the best tumor response assessment, and any disagreements were discussed to reach consensus. The documented best tumor response will be utilized to calculate and rwDCR. Meanwhile, we collected AEs (e.g., hand-foot syndrome, cutaneous rash, fatigue, hepatotoxicity, neutropenia), differential diagnoses and management strategies for AEs (such as therapeutic agents and biochemical monitoring parameters). Moreover, we extracted prices of medications for adverse event treatment, diagnostic tests, laboratory examinations, and corresponding medical consumables from the hospital information system.

### Statistical analyses

The data in this study were collected and organized using a uniformly designed, standardized Excel template. Statistical analysis was performed using R version 4.3.1 with the following analytical workflow: Categorical variables were described using frequency counts (n) and proportions (%), with between-group comparisons conducted using Mann-Whitney U test, where the test method was automatically selected based on expected frequencies. For continuous variables, normality was verified using Shapiro-Wilk test. Normally distributed data were presented as mean ± standard deviation and compared using independent samples t-test, while non-normally distributed data were described using median and interquartile range, with between-group comparisons performed via Mann-Whitney U test. All statistical analyses employed a significance threshold of α = 0.05 (two-tailed testing).

Daily medication costs before and after dose adjustments were calculated by multiplying the pre-adjustment daily dose by its unit price and the post-adjustment daily dose by its respective unit price. The daily adverse event management cost was derived by dividing the total adverse event-related expenditures (including diagnostic tests, laboratory examinations, concomitant medications, et al.) by the total treatment duration in days. AEs cost was amortized over the entire treatment period (irrespective of event timing), but hospitalization expenses and other indirect costs were not included.

We have innovatively constructed a mathematical model for alternative medication cost differential and equilibrium (Supplementary S1). A total of 1000 Monte Carlo simulation was conducted to evaluate the economic implications of adverse event rate variability between the reference medication and the alternative medication. Synthetic adverse event rates were generated from a four-parameters (2 boundary +2 shape parameters) Generalized Beta Distribution (GBD) to reflect biologically plausible bounds (Jha et al., 2016). Based on the real-world AEs incidence rates of the alternative drug regorafenib and the reference drug fruquintinib as baseline values, and referencing the AE incidence ranges from published literature, the GBD bounds were derived from pivotal trials: regorafenib any-grade AE range [0.54, 0.93] (CORRECT trial) (Li et al., 2015), fruquintinib [0.61, 0.98] (FRESCO trial) (Li et al., 2018). The initial shape parameters will be estimated through the method of moments through the mean and standard deviation from simulated data. The coefficient of variation (CV) was set to 0.1. Maximum Likelihood Estimation (MLE) was employed to estimate the GBD parameters (Myung, 2003). Convergence of MLE optimization was confirmed using the L-BFGS-B algorithm with gradient tolerance ≤ 1e-6. To account for parameter uncertainty, we integrated the Cramér-Rao Lower Bound (CRLB) to compute robust confidence intervals for the AE rates (Cavassila et al., 2001). The analysis was implemented in Python 3.8 using the Numpy and Scipy libraries. Overpricing was defined as the total daily costs exceeding the modeled price by 40%. Post hoc power analysis was assessed to check clinically meaningful differences by calculating the proportion of iterations that met this clinically margin.

The clinical pathway parameters and medication pricing data involved in this study were derived exclusively from Hospital Information System (HIS) data at Sichuan Cancer Hospital. All pricing metrics were reviewed and approved by the hospital’s pricing administration department, ensuring the authenticity and regulatory compliance of data provenance.

## Results

### Patient characteristics

**FIGURE 1:**
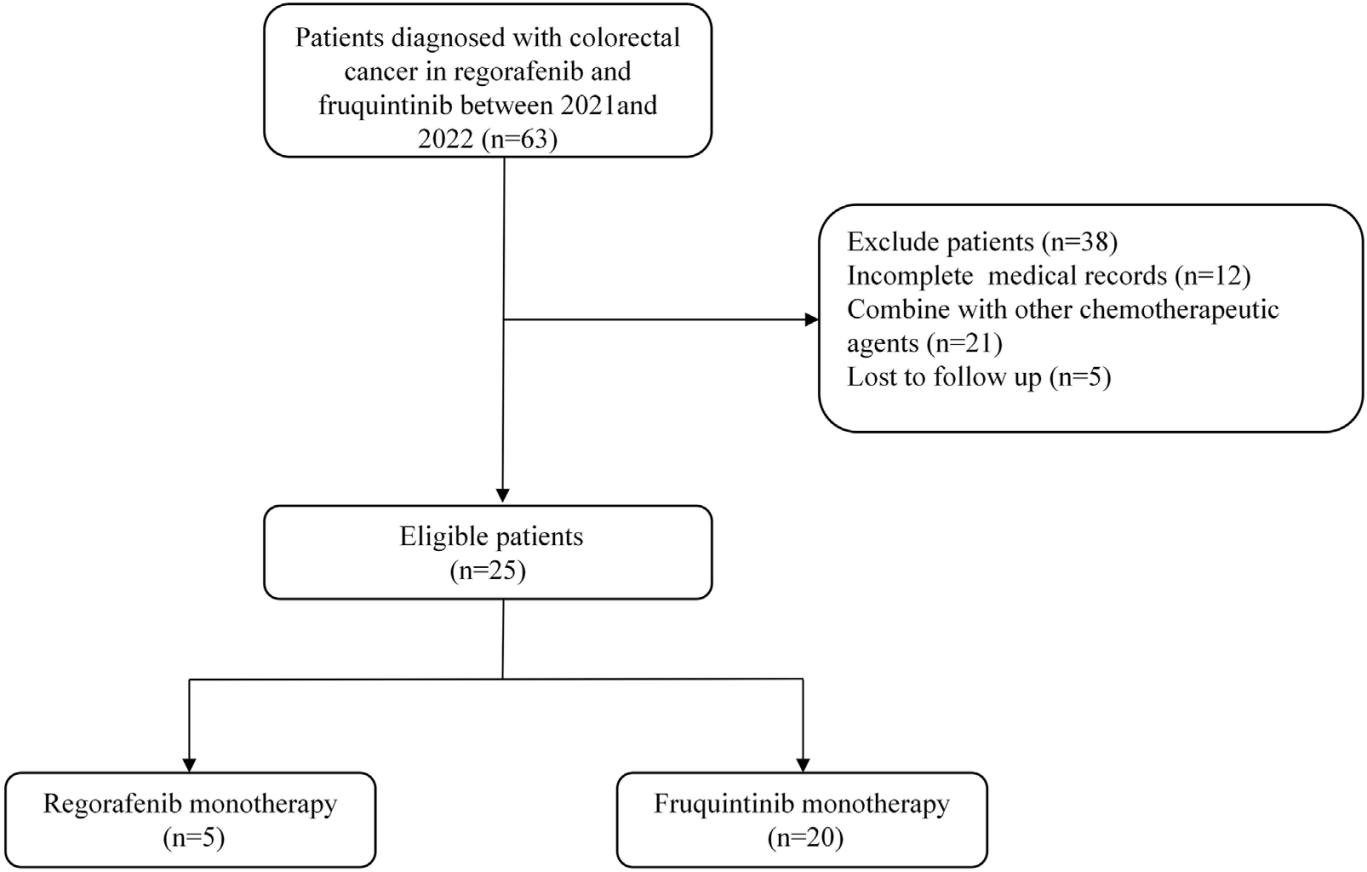
Patient Screening Flowchart.

Between January 2021 and December 2022, medical records were reviewed to identify eligible patients for this analysis. Of the 25 patients who met the inclusion criteria, the cohort comprised two treatment groups: five patients received regorafenib therapy while 20 patients were treated with fruquintinib. Figure 1 illustrates the complete patient selection process. The median follow-up times for the two cohorts were 92.0 (*IQR* 64.6-263.5) days and 97.0 (*IQR* 55.3-218.8) days, respectively. Baseline characteristic comparisons demonstrated good comparability in demographic and clinical features between the groups (Table 1). No statistically significant differences were observed in gender distribution (male: 60% vs 70%), age stratification (≥65 years: 20% vs 20%), BMI≥25 prevalence (20% vs 40%), or Eastern Cooperative Oncology Group (ECOG) performance status 0 (60% vs 70%). A trending difference in primary tumor location was noted, with the regorafenib group showing right-sided colon predominance (60%) versus rectal predominance (55%) in the fruquintinib group, though this did not reach statistical significance (p = 0.078). Metastatic burden distribution was comparable between groups (single metastasis: 40% vs 45%), with stage IV disease predominating in both cohorts (80% vs 100%). Notably, key prognostic factors including treatment lines (>95% third-line therapy) and pathological staging remained well-balanced between the groups.

**TABLE 1:** Baseline characteristics of enrolled patients.

Characteristics	Regorafenib (n = 5)		Fruquintinib (n = 20)		*p*
	n	%	n	%	
Sex					
Man	3	60.0	14	70.0	1.000
Women	2	40.0	6	30.0	
Age (years)					
<65	4	80.0	16	80.0	1.000
≥65	1	20.0	4	20.0	
BMI (kg/m^2^)					
<25	4	80.0	12	60.0	0.621
≥25	1	20.0	8	40.0	
ECOG PS					
0	3	60.0	14	70.0	1.000
1	2	40.0	6	30.0	
Primary location					
Colon	1	20.0	0	0.0	0.078
Left side	0	0.0	4	20.0	
Right side	3	60.0	5	25.0	
Rectum	1	20.0	11	55.0	
Surgery					
Yes	5	100.0	17	85.0	1.000
No	0	0.0	3	15.0	
Therapy line					
3	5	100.0	19	95.0	1.000
>3	0	0.0	1	5.0	
Stage					
IIIB	1	20.0	0	0.0	0.200
IV	4	80.0	20	100.0	
Number of metastases					
Single metastasis	2	40.0	9	45.0	1.000
Multiple metastases	3	60.0	11	55.0	

Note: BMI, body mass index; ECOG PS, eastern cooperative oncology group performance status.

### Safety and efficacy

The overall incidence of any-grade adverse events was comparable between groups at 60.0% (Table 2), however the regorafenib group demonstrated substantially higher rates of grade 3-4 events compared to the fruquintinib group (60.0% vs. 20.0%). Notable differences emerged in specific toxicities: Hepatotoxicity occurred at any grade in 40.0% (grade 3-4: 40.0%) and hand-foot skin reaction in 20.0% (grade 3-4: 20.0%) of regorafenib-treated patients, compared to corresponding rates of 50.0% any-grade (15.0% grade 3-4) hepatotoxicity and 15.0% any-grade (no severe cases) hand-foot skin reactions with fruquintinib. In contrast, fruquintinib exhibited distinct safety considerations with proteinuria (20.0% overall, 5.0% grade 3-4) and hypertension (10.0% any-grade) not observed in the regorafenib group. While these findings suggest a potentially more favorable safety profile for fruquintinib, particularly regarding severe toxicity rates, the limited sample size of the regorafenib cohort warrants cautious interpretation of between-group differences.

**TABLE 2:** Adverse event incidence rates in the regorafenib and fruquintinib groups.

Adverse events	Regorafenib (n = 5)		Fruquintinib (n = 20)	
	Any grade	Grade 3-4	Any grade	Grade 3-4
Any event	3 (60.0%)	3 (60.0%)	12 (60.0%)	4 (20.0%)
hepatotoxicity	2 (40.0%)	2 (40.0%)	10 (50.0%)	3 (15%)
Hand-foot skin reaction	1 (20.0%)	1 (20.0%)	3 (15.0%)	0
Proteinuria	0	0	4 (20.0%)	1 (5.0%)
Hypertension	0	0	2 (10.0%)	0

Table 3 presents the comparative therapeutic outcomes between regorafenib and fruquintinib. The rwDCR was 20% (1/5) in the regorafenib cohort compared to 25% (5/20) in the fruquintinib cohort, demonstrating no statistically significant difference (p = 1.000). Regarding disease progression rates, the regorafenib group showed a numerically lower proportion of progressive disease (PD) at 20% (1/5) versus 40% (8/20) in the fruquintinib group, though this difference did not reach statistical significance (p = 0.621). Of particular note, all-cause mortality rates were observed to be higher in the regorafenib-treated patients (60% [3/5] vs 35% [7/20]), but this disparity also lacked statistical significance (p = 0.358).

**TABLE 3:** Clinical outcomes in the regorafenib and fruquintinib groups.

Clinical outcomes	Regorafenib (n = 5)		Fruquintinib (n = 20)		*p*
	n	%	n	%	
rwDCR	1	20.00	5	25.00	1.000
PD	1	20.00	8	40.00	0.621
Death	3	60.00	7	35.00	0.358

Note: rwDCR, Real-world disease control rate; PD, progressive disease.

### Daily treatment costs

The regorafenib group demonstrated significantly higher mean daily costs compared to the fruquintinib group, both pre- and post-dose adjustment (pre-adjustment: ¥439.82 vs ¥259.23, P = 0.017; post-adjustment: ¥439.82 vs ¥253.71, P = 0.014). No statistically significant difference was observed in AE management costs between the groups (¥15.70 vs ¥6.25, P = 0.776), though regorafenib’s confidence intervals encompassed negative values—a finding potentially attributable to the small sample size (n = 5). When aggregating medication and AE management costs, regorafenib maintained a significantly higher total daily cost versus fruquintinib (¥455.53 vs ¥259.96, P = 0.001). Detailed data are presented in Table 4.

**TABLE 4:** Daily treatment costs in the regorafenib and fruquintinib groups.

Cost category	Regorafenib (n = 5) (¥)	Fruquintinib (n = 20) (¥)	*p*
Pre-dose adjustment daily costs	439.82 (95% CI 296.16∼583.48)	259.23 (95% CI 247.69∼270.77)	0.017
Post-dose adjustment daily costs	439.82 (95% CI 296.16∼583.48)	253.71 (95% CI 240.50∼266.91)	0.014
Daily adverse event management costs	15.70 (95% CI -20.03∼51.44)	6.25 (95% CI 1.52∼10.99)	0.776
Total daily costs	455.53 (95% CI 345.20∼565.86)	259.96 (95% CI 247.32∼272.54)	0.001

Note: CI, confidence interva; Total daily costs = Post-dose adjustment daily costs + Daily adverse event management costs.

### Result of alternative value

The mathematical model for alternative medication cost differential and equilibrium demonstrated a mean daily cost of ¥248.03 (95% confidence interval 247.98–248.09) for the alternative drug regorafenib, representing 47.35% of the real-world daily cost of ¥455.53.

72.0% of the simulation showed that the current daily price exceeded the modeled price by more than 40%, indicating a *post hoc* power of 0.72 for detecting a clinically meaningful overpricing.

## Discussion

The findings of this comparative analysis reveal critical disparities between regorafenib and fruquintinib in cost structures and safety profiles, despite comparable therapeutic efficacy. The significantly higher daily treatment costs of regorafenib (¥455.53 vs ¥259.96, P = 0.001) contrast sharply with its equivalent disease control rate (20% vs 25%, P = 1.000), reinforcing concerns about the dissociation between anticancer drug pricing and clinical value creation observed in prior studies (Vivot et al., 2017; Jiang et al., 2020; Vokinger et al., 2020). This discrepancy becomes particularly salient when considering our pharmacoeconomic model, which suggests regorafenib’s value-based alternative pricing threshold (¥248.03) aligns more closely with its therapeutic equivalence to fruquintinib. These results challenge the current pricing paradigm for third-line mCRC therapies and underscore the necessity of integrating AE management costs into value assessments.

These results challenge traditional value assessment paradigms that prioritize efficacy over safety-associated economic burdens. Our framework addresses a critical gap identified in recent ASCO Value Framework updates, which lack explicit guidance for indirect cost-value comparisons between non-comparator agents (Schnipper et al., 2016). The 53% cost differential equilibrium threshold suggests systematic overvaluation of regorafenib relative to its therapeutic equivalence with fruquintinib—a phenomenon consistent with global analyses of oncology drug pricing (Jiang et al., 2020; Ou et al., 2023; Del Paggio et al., 2017).

The divergent safety profiles observed—regorafenib’s higher incidence of severe hepatotoxicity (40% grade 3-4) versus fruquintinib’s hypertension/proteinuria risks—highlight the importance of toxicity-driven cost differentials in value calculations. While previous network meta-analyses established comparable efficacy (Zhang et al., 2020; Jing et al., 2019), our real-world data demonstrate that AE management costs disproportionately impact total treatment expenditures. The negative confidence intervals observed for regorafenib’s AE costs (n = 5) exemplify the financial uncertainty introduced by small sample sizes, emphasizing the need for larger-scale pharmacovigilance studies to refine cost projections.

Our Monte Carlo simulation using Generalized Beta Distribution parameters advances previous cost-effectiveness methodologies by incorporating biological plausibility boundaries for AE variability (de Soárez et al., 2014). Meanwhile, the GBD distribution and MLE parameter estimation have effectively addressed the uncertainty modeling challenges in adverse reaction incidence rates when working with small sample datasets. The derived cost equilibrium threshold (47.35% of current regorafenib pricing) provides actionable data for value-based pricing negotiations, addressing the “innovation paradox” where public funding drives drug discovery yet pricing fails to reflect therapeutic value (Kesselheim et al., 2015). This model aligns with emerging frameworks advocating for indication-specific pricing models in oncology (Kaltenboeck and Bach, 2018), particularly for therapies with overlapping mechanisms like VEGF inhibitors.

Study limitations include small sample size and single-center design, necessitating validation in larger cohorts. While our model focused on direct cost differentials, future iterations would benefit from incorporating EQ-5D-derived utility weights to calculate quality-adjusted life years (QALYs). We acknowledge this limitation reduces comparability with reference case cost-effectiveness analyses. Future research should incorporate QALYs metrics and societal cost perspectives to enhance model generalizability. Nevertheless, this investigation establishes a replicable methodology for value-based pricing negotiations in therapeutic classes lacking direct comparative evidence.

## Conclusion

In the absence of head-to-head trials, this cost-equilibrium framework demonstrates that fruquintinib achieves comparable disease control to regorafenib with superior safety and cost efficiency. Regorafenib’s current pricing exceeds its value-based threshold by 111%, underscoring the urgent need for value-aligned pricing reforms in oncology therapeutics. However, the interpretation of findings is constrained by the limited cohort size, which reduces statistical power to detect clinically meaningful differences in safety and efficacy outcome.

## Data Availability

The original contributions presented in the study are included in the article/Supplementary Material, further inquiries can be directed to the corresponding author.
