# Supplementary material for: Beyond efficacy parity: a novel cost-equilibrium framework for value assessment of competing third-line therapies in metastatic colorectal cancer
Source: Front Pharmacol. 2025 Aug 14;16:1606742. doi: 10.3389/fphar.2025.1606742 (PMC12390821; doi:10.3389/fphar.2025.1606742)
Supplement: Supplementary file 1 [file Supplementaryfile1.docx]

**Supplementary S1 Theoretical Framework of Mathematical Model for Alternative Medication Cost Differentials and Equilibrium**

Our proposed method for calculating the price of the substitutive medication 𝛼, denoted as 𝑃ℎ, incorporates the average incidence rate of the significant adverse reaction$M_{\alpha}$ and $M_{\beta}$:

$$P_{h}=\left( C_{\beta}\times M_{\beta}-C_{\alpha}\times M_{\alpha}+F_{\beta} \right)$$

Here C represents the disposal cost of adverse advents. ​$M_{\beta}$ is the rate of occurrence of the adverse event with the original medication 𝛽, and $M_{\alpha}$ ​ is the rate with the substitutive medication 𝛼.

Next, we expand our estimation by assuming parameters $M_{\beta}$ and $M_{\alpha}$ to follow a Generalized Beta Distribution (GBD) that can model a wide variety of shapes, with shape parameters (a, b) and (g, l) for upper bound and lower bound. Then the probability density function (PDF) denoted by pr (x|a, b, g, l) of the parameters x to be estimated according to a Beta distribution with parameters α, β, g and l is given by:

$f\left( x|a,b,g,l \right)=\frac{\left( x-g \right)^{a-1}\left( l-x \right)^{b-1}}{\left( l-g \right)^{a+b-1}B\left( a, b \right)}$.

Then, the likelihood function assuming the input parameters under GBD can be written as:

$$L_{\alpha}\left( a_{\alpha},b_{\alpha}, g_{\alpha},l_{\alpha} \right)=\prod_{i=1}^{n_{\alpha}} \frac{\left( x_{\alpha,i}-g_{\alpha} \right)^{a_{\alpha}-1}\left( l_{\alpha}-x_{\alpha,i} \right)^{b_{\alpha}-1}}{\left( l_{\alpha}-g_{\alpha} \right)^{a_{\alpha}+b_{\alpha}-1}B\left( a_{\alpha},b_{\alpha} \right)}$$

Similarly, the likelihood function for original medication 𝛽:

$$L_{\beta}\left( a_{\beta},b_{\beta},g_{\beta},l_{\beta} \right)=\prod_{j=1}^{n_{\beta}} \frac{\left( x_{\beta,j}-g_{\beta} \right)^{a_{\beta}-1}\left( l_{\beta}-x_{\beta,j} \right)^{b_{\beta}-1}}{\left( l_{\beta}-g_{\beta} \right)^{a_{\beta}+b_{\beta}-1}B\left( a_{\beta},b_{\beta} \right)}$$

We take the negative of the log-likelihood, which can then be used in maximum likelihood estimation (MLE) to establish the best estimates of 4 parameters of GBD.

The initial shape parameters will be estimated through the method of moments:

$$a=\mu\left( \frac{\mu\left( 1-\mu\right)}{\sigma^{2}}-1 \right)$$

$$b=\left( 1-\mu\right)\left( \frac{\mu\left( 1-\mu\right)}{\sigma^{2}}-1 \right)$$

Where $\mu$ is the mean and $\sigma$ is the standard deviation of rate of the significant adverse reaction from 1000 sampled simulation.

The variances of estimated parameters of GBD can be derived from the Cramér-Rao Lower Bound (CRLB) and will be used to adjust the calculation of Ph, adding a layer of statistical rigor to the pricing strategy. The CRLB provides a theoretical lower bound on the variance of unbiased estimators of a parameter. It explains the role of standard errors in modifying the adverse event rate estimates to incorporate uncertainty effectively. This approach ensures that the computed price is both statistically sound and robust against the inherent variability in parameter estimation.

Generally, for an unbiased estimator $\hat{\theta}$,

$$\text{Var}\left( \hat{\theta} \right)\geq\frac{1}{I\left( \theta\right)},$$

Where $I\left( \theta\right)$ is the Fisher information matrix that measures the amount of information that an observable random variable X carries about an unknown parameter $\theta$. $I\left( \theta\right)$ is defined to be the expected value of the negative second derivative of the log-likelihood function:

$$I\left( \theta\right)=E\left[ \left( \frac{\partial}{\partial\theta}\log f\left( X;\theta\right) \right)^{2} \right]=-E\left[ \frac{\partial^{2}}{\partial\theta^{2}}\log f\left( X;\theta\right) \right]$$

Hence, using the variances of estimators derived from CRLB, we can approximate variance of $M_{\alpha}$ and $M_{\beta}$ through Delta method.

After performing MLE, the estimated value of rate of occurrence of the adverse event with the substitutive medication and original medication $\hat{M_{\alpha}}$ and $\hat{M_{\beta}}$ can be calculated through the expected values of the GBD with corresponding estimated parameters by:

$$E[pr (x|a, b, g, l)]=g+\left( l-g \right)\frac{\alpha}{\alpha+\beta}$$

This gives:

$$\hat{M_{\alpha}}=\hat{g_{\alpha}}+\left( \hat{l_{\alpha}}-\hat{g_{\alpha}} \right)\frac{\hat{\alpha_{\alpha}}}{\hat{\alpha_{\alpha}}+\hat{\beta_{\alpha}}},$$

$$\hat{M_{\beta}}=\hat{g_{\beta}}+\left( \hat{l_{\beta}}-\hat{g_{\beta}} \right)\frac{\hat{\alpha_{\beta}}}{\hat{\alpha_{\beta}}+\hat{\beta_{\beta}}}.$$

The estimated $M_{\alpha}$ and $M_{\beta}$ from MLE update Ph formula to be:

$$\hat{P_{h}}=F_{\beta}+C_{\beta}\times\hat{M_{\beta}}-C_{\alpha}\times\hat{M_{\alpha}}$$

Finally, the 95% confidence interval of the price of the substitutive medication is given by:

$$\hat{P_{h}}\pm1.96\times\left( \sqrt{\text{Var}\left( \hat{P_{h}} \right)} \right)$$

In the above equation, we have expressed the substitutive medication accounting for both the parameters estimation and variation considerations.
